# Successful treatment of acute oral copper sulfate poisoning: a case report

**DOI:** 10.3389/fphar.2026.1879318

**Published:** 2026-08-06

**Authors:** Yingxin Zhao, Ruifang Li, Chenguang Wang, Chen Li, Lijun Wang

**Affiliations:** Tianjin Medical University General Hospital, Tianjin, China

**Keywords:** chelating ligand, copper sulfate, poisoning, serum copper, sodium dimercaptopropane sulfonate, urine copper

## Abstract

The incidence of copper sulfate poisoning varies across different regions. It is relatively rare in Western countries but more prevalent in South Asian nations, accounting for approximately 40% of all heavy metal poisoning cases. The majority of affected individuals are rural residents, with a mortality rate ranging from 14% to 36%. Copper sulfate poisoning often leads to gastrointestinal corrosion, intravascular hemolysis, hemolytic anemia, methemoglobinemia, and acute multi-organ dysfunction, often resulting in death due to delayed treatment. In June 2024, we successfully managed a case of acute oral copper sulfate poisoning through early interventions, including gastric lavage and sodium dimercaptopropane sulfonate (Na-DMPS) for copper chelation, resulting in the patient’s recovery and discharge after 1 week.

## Case report

A 16-year-old male patient had a height of 180 cm and weight of 60 kg, with a body mass index (BMI) of 18.5 kg/m^2^ presented to the hospital 1 h after ingesting 100 mL of a homemade liquid containing pentahydrate copper sulfate (concentration 38%). The patient had a history of anxiety and depressive states, treated with fluvoxamine maleate and aripiprazole. The patient developed nausea, vomiting of bile-green gastric contents, and periumbilical abdominal pain approximately 5–6 min after ingestion, with no hematemesis, melena, or hematuria reported. Self-induced vomiting was performed half an hour before emergency admission. Upon admission, Gastric lavage was performed approximately 1 h after ingestion. And other immediate symptomatic treatments were administered including, administration of egg white and milk to dilute toxic substances for protecting the gastric mucosa, omeprazole for acid suppression, fluid replacement, and glutathione at a dose of 2.4 g/d by intravenous infusion for 3 consecutive days to counteract copper-induced oxidative stress and hepatic injury (Sun, 2020). Blood tests including complete blood count, liver and kidney function, electrolytes, cardiac enzymes, and troponin showed no significant abnormalities. Toxicological analysis revealed a serum copper concentration of 3775.0 ng/mL (reference interval: 800–1,900 ng/mL). The patient continued to complain of abdominal pain, most notably around the umbilical region. After the toxicological results became available, sodium dimercaptopropanesulfonate (Na-DMPS) was administered at a daily dose of 0.125 g for three consecutive days as copper chelation therapy. Continuous monitoring over 3 days showed a decreasing trend in serum and urine copper levels (Table 1). A follow-up examination 3 days later showed normalization of urine copper and there were no significant changes in hemoglobin levels, liver, or kidney function (Table 2). The patient reported no discomfort and was discharged after 1 week. Ten days post-discharge, follow-up revealed no reports of symptoms, and retesting showed serum copper, urine copper, complete blood count, and liver and kidney function all within normal ranges (Table 2) (Chandra et al., 2024; Nastoulis et al., 2017).

**TABLE 1:** Changes in serum copper and urine copper with Na-DMPS therapy.

Serum/urine copper levels	On admission	Day l	Day 2	Day 3	Day 4	Day 5	Day l6	Reference range
Serum copper (ug/L)	3775	​	735.6	752.6	704.8	733.6	859.6	800∼1,900
Urine copper (ug/24 h)	​	​	401.3	155.7	122.7	57	22.3	15∼16
Na-DMPS dosage	0.125 g	0.125 g	0.125 g	​	​	​	​	​

**TABLE 2:** Summary of laboratory test results during hospitalization.

DATE	WBC (*10^9^/L)	RBC (*10^12^/L)	HB (g/L)	PLT (*10^9^/L)	ALT (U/L)	AST (U/L)	ALKP (U/L)	LDH (U/L)	TBIL (umol/L)	CREA (umol/L)
On admission	10.94	5.16	159	211	22	26	201	150	13.1	56
Day l	6.69	4.79	147	203	14	23	194	136	23.9	54
Day 6	6.29	4.78	149	218	11	14	202	140	16.1	65
Day l6	5.36	4.82	150	231	9	23	179	158	28.9	63
Reference range	4.4∼11	4.5∼5.9	129∼172	150∼407	7∼43	12∼37	38∼126	120∼250	3∼22	52∼101

## Discussion

Copper sulfate, also known as blue vitriol, is a white or grayish white powder. Its aqueous solution is weakly acidic and exhibits a blue color. It is primarily used in agricultural production as an insecticide, disinfectant, feed additive, and soil amendment. Copper sulfate is also utilized in various industries, including aquaculture, food processing, and healthcare sectors. In China, compared to poisoning by other heavy metals such as lead, mercury, and arsenic, copper sulfate poisoning is relatively uncommon. Acute copper sulfate poisoning typically occurs due to gastrointestinal exposure, but it can also be seen in cases of respiratory and lung damage from inhaling copper dust, local skin and mucous membrane damage or necrosis from contact with high concentrations of copper compounds, and severe systemic poisoning from extensive contact with copper sulfate on wounds (Nastoulis et al., 2017). Ingesting more than 0.5 g can cause reflex vomiting, and exceeding 1 g can lead to poisoning symptoms. The median lethal dose (LD50) ranges from 10 to 20 g (Park et al., 2018).

**FIGURE 1:**
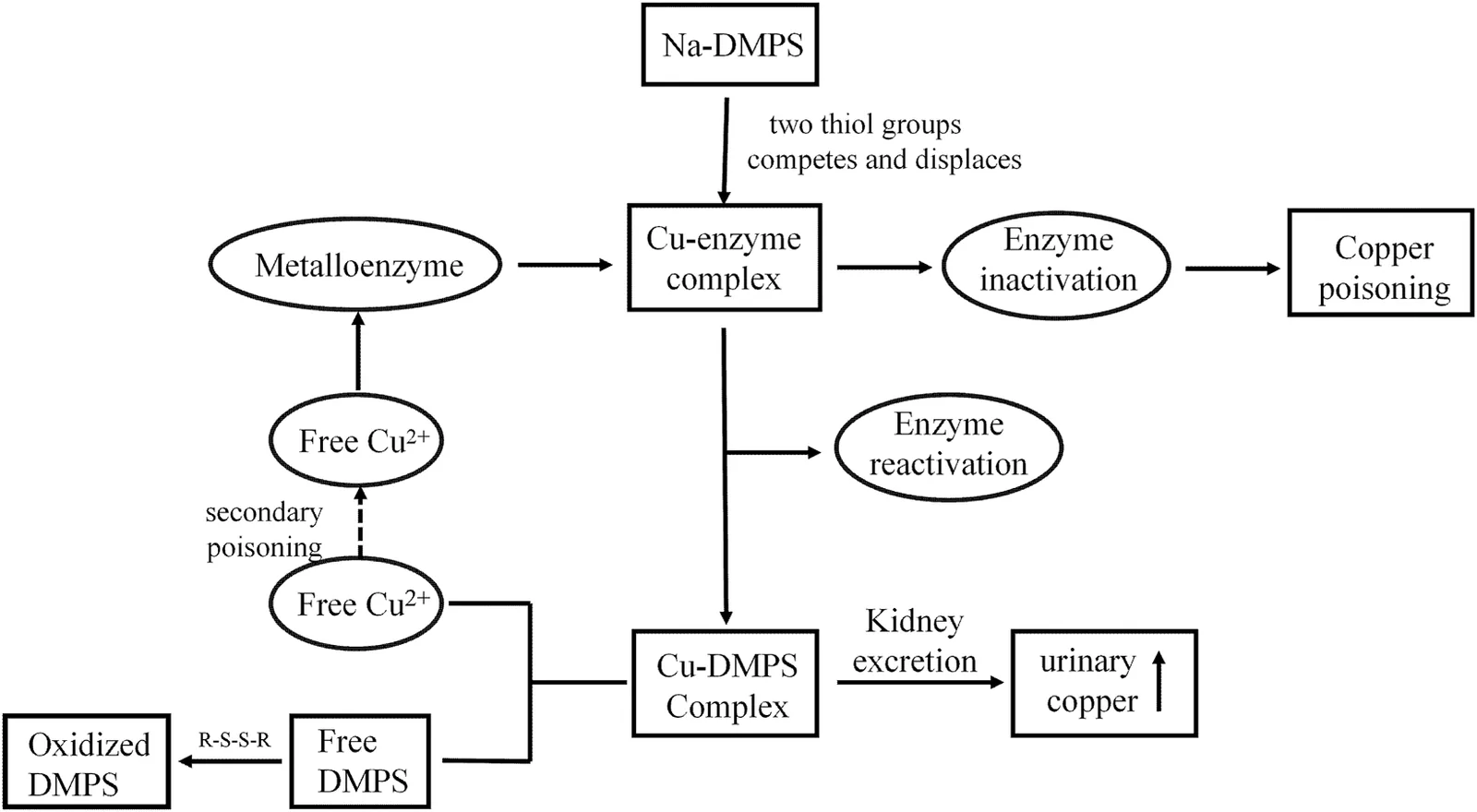
Na-DMPS for the treatment of acute copper sulfate poisoning mechanisms.

Acute copper sulfate poisoning commonly presents with symptoms such as nausea, vomiting, and abdominal pain. In severe cases, it may lead to hematemesis, melena, ulcers, or even perforation. Additionally, other complications may arise, including acute liver failure, intravascular hemolysis, rhabdomyolysis, methemoglobinemia, and acute kidney injury (Rodgers, 1997),Furthermore, heart failure, arrhythmia, epileptic seizures, coma, and even death may also occur (Saravu et al., 2007). Currently, the treatment for acute copper sulfate poisoning primarily includes: inducing emesis, gastric lavage, protecting the gastric mucosa, balancing body fluids, stabilizing the internal environment with supportive care, chelation therapy, and blood purification (Nelson et al., 2019; Franchitto et al., 2008; Du and Mou, 2019). Na-DMPS (sodium 2,3-dimercaptopropane-1-sulfonate; also known as Dimaval®) is available as both injectable (250 mg/5 mL ampoule) and oral (100 mg capsule) formulations. It is a water-soluble chemical analog of dimercaprol (BAL) with lower toxicity and is widely used as a broad-spectrum heavy metal chelator for poisoning with mercury, arsenic, chromium, bismuth, copper, antimony, and other metals (Du and Mou, 2019). In the treatment of mercury poisoning, the efficacy of Na-DMPS as a mercury-chelating agent has been well established. The standard regimen is: 0.5–0.75 g/d intramuscularly in 2–3 divided doses for several days initially, followed by 0.125 g intramuscularly twice daily for 3 days, then a 4-day rest, with a 7-day course as one cycle (Sun, 2020). Na-DMPS was later discovered to be effective in the treatment of Wilson’s disease and has gradually been used for copper poisoning treatment (Dong et al., 2021; Aposhian et al., 1995; Cao et al., 2015; Tominaga et al., 2021). While DMPS has been extensively used in Eastern Europe and Asia for decades, its availability varies by region; in some countries, it may be obtained through poison control centers or as an orphan drug for specific indications. We have noted that clinicians should be aware of local availability and consider consulting regional poison information centers when managing acute copper poisoning cases. The efficacy of chelating agents in treating acute copper sulfate poisoning is limited and results varyThere is no standardized protocol for the optimal choice of chelating agents and their duration of use. The dosage used is often extrapolated from the mercury poisoning regimen, with considerable inter-individual variability in treatment response.The patient ingested 100 mL of 38% copper sulfate pentahydrate solution, delivering 24.32 g copper sulfate—a dose surpassing lethal levels. Owing to the inherent emetic effect of copper sulfate, reflex vomiting occurred minutes after ingestion, and gastric lavage administered within 1 h of exposure markedly reduced systemic toxin uptake. The patient was hospitalized with serum copper concentration of 3,775 ug/L, significantly higher than the normal range (800–1,900 ug/L). Following gastric lavage to remove the toxin, fluid resuscitation, and early administration of a sufficient dose of the chelating agent Na-DMPS for copper excretion, the patient showed a significant increase in urine copper excretion and a rapid reduction in serum copper to normal levels. There were no severe gastrointestinal complications or organ dysfunction, and the prognosis was favorable. Accordingly, for patients with acute copper sulfate intoxication, early gastrointestinal decontamination is conducive to reducing systemic toxin absorption. Adjunctive chelation therapy can substantially enhance urinary copper excretion, indicating that Na-DMPS serves as an effective antidotal regimen for eliminating excess copper in such poisoning cases. The main mechanism of action is that Na-DMPS, with its two thiol groups, can complex with copper ions to form non-toxic, less dissociative complexes excreted in urine. Dimercapto compounds have a strong affinity for metals and can displace metals that are already bound to enzymes, thereby restoring enzyme activity. Due to the partial dissociation of the complexes formed by dimercapto drugs and their slow excretion, if they dissociate, the dimercapto compounds can be rapidly oxidized, and free metals can still cause poisoning (Figure 1). Therefore, for acute metal poisoning, chelating agents should be administered repeatedly and in sufficient doses. Additionally, chelating agents themselves can affect renal function, and there have been reports of chelating agent therapy causing organ damage or even death. Therefore, renal function monitoring is necessary during their administration (Rodgers, 1997; Sinkovic et al., 2008)^.^ Sufficient early gastrointestinal decontamination prevented severe complications in this patient; thus, the 5 mg/kg loading dose of Na-DMPS was withheld, and a 2.5 mg/kg maintenance dose was administered for 3 days (Franchitto et al., 2008). Serum copper returned to normal on day 4. Serial complete blood counts, liver function tests, and renal function tests at admission, on day 1, and on day 6 were unremarkable, with full resolution of gastrointestinal symptoms and no new complications. As an effective heavy metal chelator, Na-DMPS shows outstanding antidotal efficacy for mercury poisoning and is also active against arsenic, chromium, bismuth, copper, and antimony toxicoses. However, only case reports regarding its use in acute copper sulfate intoxication have been published, and relevant randomized controlled trials are still lacking (Chandra et al., 2024; Andersen and Aaseth, 2016). Additional rigorous clinical research is required to accumulate evidence for this treatment regimen.

## Summary

The favorable outcome likely reflects multifactorial contributions—early emesis, gastric lavage, aggressive supportive care, and possibly limited systemic absorption—rather than DMPS alone. In a single uncontrolled case, the efficacy of DMPS in severe acute copper sulfate poisoning cannot be established. Its use requires individualised risk–benefit assessment, preferably in consultation with a clinical toxicologist, with vigilant monitoring for adverse effects. Well-designed studies are needed to define its role. Meanwhile, early supportive care—fluid resuscitation, electrolyte correction, management of haemolysis and renal impairment, and gastrointestinal decontamination when indicated—remains the mainstay of management.

## Data Availability

The original contributions presented in the study are included in the article/supplementary material, further inquiries can be directed to the corresponding author.

## References

[B1] Andersen O. Aaseth J. (2016). A review of pitfalls and progress in chelation treatment of metal poisonings. J. Trace Elem. Med. Biol. Organ Soc. Minerals Trace Elem. (GMS) 38, 74–80. 10.1016/j.jtemb.2016.03.013 27150911

[B2] Aposhian H. V. Maiorino R. M. Gonzalez-Ramirez D. Zuniga-Charles M. Xu Z. Hurlbut K. M. (1995). Mobilization of heavy metals by newer, therapeutically useful chelating agents. Toxicology 97 (1-3), 23–38. 10.1016/0300-483x(95)02965-b 7716789

[B3] Cao Y. Skaug M. A. Andersen O. Aaseth J. (2015). Chelation therapy in intoxications with mercury, lead and copper. J. Trace Elem. Med. Biol. 31, 188–192. 10.1016/j.jtemb.2014.04.010 24894443

[B4] Chandra A. Ansar M. Chatterjee A. Dasgupta S. (2024). Fatal intravascular haemolysis due to copper sulfate poisoning: insights and literature review. BMJ Case Rep. 17 (12), e260160. 10.1136/bcr-2024-260160 39950634

[B5] Dong Z. P. Shao H. Y. Zhao Y. X. (2021). A case of acute copper sulphate poisoning and literature review. Zhonghua Lao Dong Wei Sheng Zhi Ye Bing Za Zhi 39 (11), 871–873. 10.3760/cma.j.cn121094-20200714-00408 34886653

[B6] Du Y. Mou Y. (2019). The role of plasmapheresis in treating lethal cupric sulfate poisoning. Am. J. Med. Sci. 357 (4), 338–342. 10.1016/j.amjms.2018.11.014 30638603

[B7] Franchitto N. Gandia-Mailly P. Georges B. Galinier A. Telmon N. Ducassé J. L. (2008). Acute copper sulphate poisoning: a case report and literature review. Resuscitation 78 (1), 92–96. 10.1016/j.resuscitation.2008.02.017 18482790

[B8] Nastoulis E. Karakasi M. V. Couvaris C. M. Kapetanakis S. Fiska A. Pavlidis P. (2017). Greenish-blue gastric content: literature review and case report on acute copper sulphate poisoning. Forensic Sci. Rev. 29 (1), 77–91. Available online at: https://pubmed.ncbi.nlm.nih.gov/28119268/. 28119268

[B9] Nelson L. S. Howland M. A. Lewin N. A. Smith S. W. Goldfrank L. R. Weisman R. S. (2019). Goldfrank's Toxicologic Emergencies. 11th ed. (New York, NY: McGraw-Hill Education), 1570–1578.

[B10] Park K. S. Kwon J. H. Park S. H. Ha W. Lee J. An H. C. (2018). Acute copper sulfate poisoning resulting from dermal absorption. Am. J. Ind. Med. 61 (9), 783–788. 10.1002/ajim.22892 30053322

[B11] Rodgers G. C. (1997). Ellenhorn’s medical toxicology: diagnosis and treatment of human poisoning. J. Am. Med. Assoc. 278 (14), 1201. 10.1001/jama.1997.03550140095052

[B12] Saravu K. Jose J. Bhat M. N. Jimmy B. Shastry B. A. (2007). Acute ingestion of copper sulphate: a review on its clinical manifestations and management. Indian J. Crit. Care Med. 11 (2), 74–80. 10.4103/0972-5229.33389

[B13] Sinkovic A. Strdin A. Svensek F. (2008). Severe acute copper sulphate poisoning: a case report. Arh. Hig. Rada Toksikol. 59 (1), 31–35. 10.2478/10004-1254-59-2008-1847 18407869

[B14] Sun C. Y. (2020). Practical Encyclopedia of Acute Poisoning. 2nd ed. (Beijing: People’s Medical Publishing House).

[B15] Tominaga T. Shimomura S. Tanosaki S. Kobayashi N. Ikeda T. Yamamoto T. (2021). Effects of the chelating agent DTPA on naturally accumulating metals in the body. Toxicol. Lett. 350, 283–291. 10.1016/j.toxlet.2021.08.001 34371142

